# 

**Published:** 1901-01-15

**Authors:** 


ADDRESSES AND PAPERS.

Address of President, L. L. Barber, 29.
Anesthesia, continued, with Nitrous Oxid, W. A. Heckard, 387.
Anesthetics, Administration of, S. O. Goldman, 7.
Basal Temperament, Relative to Prosthesis, B. J. Cigrand, 595.
Bridge Splint for Pyorrhea Cases, W. B. Ames, 545.
Cavity Preparation, C. N. Johnson, 71.
Cone for Root Filling, Theo. F. von Beust, 199.
Critical Periods in the History of Human Teeth, C. N. Johnson, 394.
Crown and Bridge Work, modern methods, J. L. Young, 365.
Dental Antiseptics and Disinfectants, B. L. Kesler, 487.
Dental Pulp, James G. Palmer, 234.
Dentists in U. S. Army, J. S. Marshall, 550.
Diagnosis, Importance of, H. H. Harrison, 188.
Diagnosis and Treatment of Empyemia of Maxillary Sinus, W. H. G. Logan, 458.
Epilepsy, its Dental Aspect, H. C. Brown, 197.
Eruption of the Teeth, T. E. Constant, 124.
Etiology of Disease, C. L. Hungerford, 456.
Etiology, Physiology and Prophylaxis, J. D. Patterson, 452.
Extension for Prevention, Wm. H. Truman, 302.
Extension for Prevention, C. M. Wright, 109.
Facial Restoration, J. A. Heidbrink, 249.
"Ghosts," Burt Abell, 220.
Gum Borders and Sockets, diseases of, C. M. Wright, 440.
Hygiene of the Mouth, B. L. Kesler, 383.
Ideal Reporter, C. M. Wright, 217.
Impacted Third Molars, J. W. Shaw, 1.
Implanted Teeth and Their Attachments, W. C. Barrett, 463.
International Dental Federation, E. C. Kirk, 561.
Limits of Dental Therapeutics, E. L. Clifford, 608.
Little Ones in the Dental Chair, C. R. Rowley, 617.
Maxillary Sinus, Diseases of, Thos. L. Gilmer, 271.
Memorial Address, Dr. Geo. W. Keeley, C. M. Wright, 415.
Miscellaneous Transactions of National Dental Association, 574.
Models, E. C. Moore, 163.
Moral Side of Dentistry, Geo. W. Clapp, 291.
Moral Side of Dentistry, second paper, Geo. W. Clapp, 433.
Mucus Membrane, Lesions of, A. W. Harlan, 201.
Neurasthenia, Dental, W. H. Whitslar, 355.
Neurology, Dental, W. H. Whitslar, 55.
Neurosis, Its Chemical Aspect, J. S. Cassidy, 36.
Odontomes, Thomas L. Gilmer, 541.
Opening Constricted Root Canals, F. T. Hays, 96.
Orthodontia, C. B. Blackmarr, 325.
Oxyphosphate Cement, Herman Fleck, 136.
Palatal Defects, and their Treatment, T. W. Brophy, 246.
Pathology in Dental Practice, G. W. Cook, 521.
Porcelain as an Art in Crown Work, F. J. Capon, 379.
Porcelain Crown Work, H. J. Goslee, 467.
Porcelain, The Use of, John J. Green, 226.

Proceeding of National Dental Association, 449.
Prosthetic Dentistry, Geo. H. Wilson. 168.
Reflex Irritation, J. Taft, 194.
Secret Preparations, S. G. Perry, 299.
Syphilis, as Seen by the Dentist, N. D. Coons, 16.
Syphilis, a Case in Practice, L. M. James, 25.
Treatment of Root Canals, Wm. E. Harper, 405.
When and How may we Destroy the Dental Pulp, N. S. Hoff, 280.

PARAGRAPHS.

Adapting Gold Foil to Cervical Margin, F. W. Stephan, 583.
Amalgam and Gold Filling, J. W. Clark, 474.
Antiseptic Floss Toothpick, J. W. Cowan, 529.
Bad Business Methods, 420.
Banded Logan Crown, W. H. Gensley, 142.
Bridge Attachment, Concealed, H. M. Kirke, 185.
Bridge Dummies, J. K. Smith, 143.
Carmichael's Crown and Bridge Work, 581.
Carious Tooth in Dermoid Cyst, J. R. Calahan, 586.
Carved Cusps, J. E. Nyman, 529.
Cast Aluminum, W. O. Hulick, 143.
Chloroform, 13.
Clinics at National Dental Association, S. H. Guilford, 578.
Crown and Bridge Mounting, H. J. Goslee, 167.
Dental Education, Its Status, 31.
Dental Society, it's Advantages, 34.
Dento-facial Deformities, C. S. Case, 530.
Disinfecting the hands, 184.
Engine Disk Guard, 586.
English Dental Companies, 95.
Etiology of Degeneracy of Human Teeth, R. B. Adair, 623,
Foreign Dental Schools, their Standards, 516.
Foreigh Relations, Committee Report, 511.
Formaline, E. C. Kirk, 305.
Food of Prehistoric man, 483.
Fraudulent Dental Degrees, 513.
Gelatin as a Hemostatic, 252.
Gold Blindness, 307.
Gold and Felt Foil, L. T. Canfield, 146.
Gold Filling, with Smooth Plugger Points, J. B. Bauman, 140.
Gold Foil Root Fillings, I. Douglas, 585.
Gold and Tin Filling, J. R. Callahan, 141.
Hemophilia causes Death, 193.
Hemorrhage after Extracting, 100.
Improved Tin Foil, H. L. Ambler, 528.
Inlay Matrix, W. T. Reeves, 232.
Illinois State Board of Examiners, 502.
Iodoform Odors, to remove, 123.
Mallet Blow, to deaden, 35.
Mat Gold, J. B. Vernon, 585.
Matrix in Filling, H. M. Semans, 140.
Matrix modified, C. T. Whinnery, 144.
Matrix for Seamless Crowns, J. H. Prothro, 147.
Miller's Contouring Pliers, 582.
Mouth Toilet of Orientals, 202.
Mouth Wash, 351.
Music and Anesthesia, 305.
Naphthol Alpha and Beta 151.
New Appliances, W. A. V. Price, 145.
New Metal for Cast Plates, R. C. Brophy, 581.
Nitrous Oxide and Air, 12.
Nitrous Oxide and Carbon Dioxide, 11.
Observations on the Motions of the Mandible, C. Tomes, 317.
Parker Swaging Machine, B. H. Lee, 584.
Plaster of Paris and Pumice for Impressions, 6.
Porcelain body Colors, W. S. Taggart, 229.
Porcelain Crown, C. E. Redmond, 529.

Porcelain Inlays, D. W. Clancy, 141.
Porcelain Inlays, E. E. Rees, 473.
Pulp Digestion, A. W. Harlan, 132.
Pyrozone, to rust impacted nerve broaches, 139.
Removable Beaks for Extracting Forceps, C. V. Vignes, 584.
Removing Excess Cement in setting Crowns, S. D. Ruggles, 474.
Removing Plaster from Vulcanite Plates, 100.
Report of Meeting of International Dental Federation, 567.
Root Dryer, H. P. Carlton, 475.
Rubber Dam in s tting Logan Crowns, J. E. Boyd, 142.
Sandvig's Swaging Apparattus, I. Ottensen, 584.
Scalers, new forms, C. R. Butler, 528.
Sensitive Teeth, C. C. Harris, 48.
Soft Foil Contour Fillings, G. S. Junkerman, 142.
Sponge, death from inhaling, 200.
Swaging Metal Plates, E. L. Patch-
The Use of Silver Foil, L. S. Chilcott, 627.
in, 144.
Tooth Powder, 349.
Tooth Wash, 350.
Truing Carborundum Wheels, G. B. Perry, 473.

### INDENTS.

Abscess with Necrosis, 215.
Acetic Acid as a Germicide, 213.
Adapting Metal Backs to Porcelain Facings, 37.
Advisability of Administering Anesthetics, 323.
Amalgam Fillings, to remove, 161.
Anesthetic Mixture, 377.
Aristol in a pulp varnish, 538.
Antrum, is it ever divided? 538.
Arrest of hemorrhage after extracting, 105.
Artistic Dentures, 431.
Banded Crown Setting, 216.
Bleaching Teeth with Compressed Air, 214.
Cavity Preparation, 323.
Chapped Hands, 213.
Cleaning the Teeth, 430.
Chloretone in Pyorroea, 107.
Cocaine Solutions, 484.
Cocaine Antidote, 215.
Collodian Model Varnish, 214.
Combination Amalgam and Cement Filling, 322.
Copper Amalgam, for deciduous teeth, 215.
Counter Irritant, 266.
Crowns for First Permanent Molars, 322.
Disinfecting a Putrid Canal, 266.
Emergency Crowns, 270.
Ethics, 268.
Extracting with the Fingers, 484.
Fermentative Diseases of Infants, 485,
Filling for Immunity, 539.
Fixing Loose Teeth, 431.
Glycerine Solution of Formaline, 376.
Gold Inlay Matrix, 484.
Gutta Percha, filling, finishing, 213.
Gutta Percha for Exposed Margins, 324.
Hemophilic Patients, 106.
High Priced Instruments, 267.
Ideal Base Plate, to renew, 159.
Jack Screw, 376.
Kowarko's Cement, 159 and 160.
Logan Crown, to set, 593.
Loosening of the Teeth, 538.
Making Inlay Matrix, 268.
Mallet, 213.
Molar Tooth in Orbital Plate, 214.
New Form of Tooth Brush, 484.
Nirvanin, 107.
Ontario's Four Years Course in Dentistry, 377.
Papain Paste, 161.
Peridental Membrane and Crowns, 376.
Platinum Inlay Matrices, 160.
Polishing Aluminum Plates, 269.
Porcelain Inlays, 106.
Porcelain, to prevent becoming porous, 162.
Practicable Porcelain inlays, 107.
Preparation for Crowning, 485.

Proper Sphere for the All Gold Crown, 267.
Pulp Anodyne, 267.
Pulp Capping, 432.
Pulverized Soapstone, 484.
Removal of Devitalized Pulps, 376.
Repairing Broken Models, 378.
Right Attitude toward Patients, 594.
Scaling Sensitive Teeth, 105.
Setting Crowns with Gutta-Percha, 539.
Smoker's Gingivitis, 538.
Sodium Dioxide as a Bleacher, 430.
Sodium Peroxide, 107.
Sodium Peroxine and Sulphuric Acid, 486.
Soft Solder Powder, 593.
Solder Alloy, 213.
Suprarenal Capsule, 214.
Tactile Impressions, 538.
Tannocreosoform, 214.
Tin and Gold Coloration, 161.
Trade Journals, 269.
Transforming Logan Crowns, 266.
Treatment of Pulp Canal after Pulp Removal, 105.
Toothache, new cure, 376.
Toothache Wax, 322.
Tooth Powder, 105.
Upper Fourth Molar, 159.
Vacuum Chamber, 106.
Varnish for Cement Fillings. 266.
Young Man in Dentistry, 378.

### EDITORIAL.

Administration Difficulties, 424.
Alumini Association, Louisville Dental College, 312.
American Journal of Dental Science, dead, 206.
Asbestos Wig, 154.
Biological Study of Odontology, 478.
Chicago Dental College Alumni Clinic, 51.
Cocaine Injection Fatal, 205.
Dental Register for 1901, 49.
Dental Brief, 53.
Dental Cosmos, 101.
Dental Diploma Traffic, 477.
Dental Era, 633.
Disreputable Practitioners in Ohio, 204.
Dramatic Incident at Milwaukee, 425.
Fifty-fifth Volume, 632.
Encomiums of H. J. McKellops, 372.
Examination Subjects for Army Surgeons, 155, 203.
Foreign Relations Committee, 54.
Four Year College Course, 422, 532.
Future Tri-State Conventions, 311.
Grading Dental Colleges, 479.
Hunt, Dr. G. E., honored, 312.
Indiana Dental Journal, death, 51.
Institute of Dental Pedagogics, 50, 590.
International Dental Journal, 53.
Johnson, Dr. C. N., and Detroit Dental Society, 152.
Keely, Dr. George, memorial tablet, 313.
London Dental Hospital, 262.
Mahoning Valley Dental Society, 312.
Manila Dental College, 206.
Medical Department University of Penn., new building, 312.
Memorial Tablets, Hayden and Harris, 312.
New Dean, Dental Department Ohio Medical University, 425.
Mew Editor Dominion Dental Journal, 207.
"Now is the Time to Subscribe," 633.
Officers Elected by Ohio State Dental Society, 54, 155.
Ohio State Dental Society Meeting, 589.
Permanent Fillings, 153.
Price of Artificial Teeth, 101.
Professional Matters, 588.
Program for St. Louis World's Fair Meeting, 589.
Proximal or Proximate, 371.
Request for Names of Members of State Examining Boards, 533.
Robie, death of Col. John E., 54.
Roentgen Rays, 475.
S. S. White Dental Manfg. Co., 103.
Southwestern Michigan Dental Society, 155.
Suction Chambers, 102.

Sun-down Dental Colleges, 423.
Therapeutics, classification for, 102.
Too Many Papers, 372.
Transactions National Dental Association, 479.
Tri-State Dental Convention, 154, 259, 308.
What Made the Tri-State Meetings So Popular, 311.

ANNOUNCEMENTS.

American Society of Orthodontists, 481.
American Dental Society of Europe, 321.
Chicago Dental Society Officers, 265.
Detroit Dental Society, 321.
Illinois Dental Society Officers, 321.
Illinois Dental Society Meeting, 207.
Illinois, Kentucky and Indiana Tri State Meeting, 212.
Kentucky State Society, 212.
Michigan State Dental Association Officers, 429.
Missouri State Dental Society, 265.
National Association Dental Examiners, 375.
National Association of Dental Faculties, 375.
National Dental Association Officers, 482.
National Dental Association, 320, 375.
New Jersey State Society, 212, 264.
Northern Ohio Dental Society, 265.
Officers National Association of Examiners, 482.
Officers National Association of Faculties, 481.
Ohio State Board of Examiners, 265, 590.
Oklahoma Dental Examiners, 212.
Pennsylvania Board of Examiners, 212.
Program of Institute of Dental Pedagogics, 591.
Program of Ohio State Dental Society, 590.
Program Tri-State Convention, 253.
Southern Branch National Dental Association, 265.
Tri State Dental Convention, 211.
West Virginia Dental Examiners, 212.

BIBLIOGRAPHY.

American Text-Book on Operative Dentistry, 374.
Barrett's Oral Pathology and Practice, 428.
Cryer's Internal Anatomy of the Face, 636.
Dental Electricity, 426.
Dental Medicine, 480.
Essig's Dental Metallurgy, 374.
Gray's Anatomy, 637.
Interrogations in Dental Metallurgy, 373.
Marshall's Operative Dentistry, 373.
Oral Surgery, 373.
Poems of the Farm, 263.
Principles and Practice of Filling Teeth, 159.
Talbot's Irregularities of the Teeth, 638.
Questions on Medical Subjects, 319.

OBITUARY.

Charles E. Esterly, 534.
T. A. Long, 634.
H. J. McKellops 263, 316.
W. H. Morgan, 313.
C. F. Stipp, 635.
Wm. Taft, 634.
J. G. Van Marter, 635.
Nelson W. Williams, 314.

AUTOGRAPHIC.

Abell, B., 220, 339.
Albrecht, Dr., 457.
Adair, R. B., 623.
Allan, C. F., 484.
Allis, D. H., 5.
Ambler, H. L., 364, 528.
Ames, W. V. B., 545, 550.
Andrews, R. R., 242.
Arnold, Otto, 65.
Barber, L. L., 29.
Barnes, H., 47, 361.
Barnett, W. M., 105.

Barrett, W. C., 455, 462, 463, 544.
Bauman, J. B., 140.
Black, G. V., 105, 244, 267, 376, 466, 539.
Blackmarr, C. B., 325, 334, 337.
Bogue, E. A., 105, 245, 466, 550.
Booth, Dr., 362.
Boyed, J. E., 142.
Boozer, D. L., 100.
Brewster, R. C., 107.
Brockway, A. H., 161.
Broomell, I. N., 239, 268.
Brown, G. V. I., 460.
Brown, H. C. 197.
Brophy, T. W., 246.
Brophy, R. C., 581.
Burke J. J., 215.
Burkhart, A. P., 266.
Butler, C. R., 67, 121, 196, 393, 528.
Callahan, J. R., 81, 141, 586.
Canfield, L. T., 146.
Capon, F. J., 106, 379, 485.
Carlton, H. P., 475.
Case, C. S., 530.
Cassidy, J. S., 36 48, 392.
Chilcott, L. S., 627.
Cigrand, B. J., 595.
Clancy, D. W., 48, 141.
Clark, J. N., 474.
Clark, Harold, 105, 376.
Clapp, Geo. W., 291, 433.
Clifford, E. L., 608.
Codding, F. H., 336.
Collins, Dr., 290.
Cook, W. A., 402, 461, 521.
Coons, N. D., 16.
Cormany, J. W., 458.
Cowan, J. W., 529.
Constant, T. E., 124.
Cravens, J. E., 447.
Cromar, John, 159.
Cryer, M. H., 159, 538.
Custer, L. E., 462.
Daboll, 266.
Donnally, W., 560.
Douglas, I., 361, 393, 585.
Duffield, Dr., 484.
Ebersole, Dr., 393.
Emory, Dr., 364, 392.
Essig, F. H., 333, 339.
Faxon, Dr., 5.
Ferris, Dr., 107.
Fillebrown, Thos., 464, 543.
Finley, M. F., 452, 544.
Fleck, Herman, 132.
Fletcher, M. H., 45.
Franck, Johann, 106.
Freeman, S., 214.
Fowler, S. M., 414.
Gallie, Don M., 403.
Genseley, 142.
Gilmer, T. L., 271, 342, 461, 541, 545.
Goldman, S. O., 7.
Goslee, H. J., 107, 167, 386, 467.
Green, John J., 226.
Grove, C., 215.
Guilford. S. H., 565, 548, 578.
Hamilton, H. F., 484.
Harlan, A. W., 132, 161, 201, 430, 431, 538.
Harper, J. G., 484.
Harper, W. E., 405, 414, 415.
Harrison, H. H., 120, 188, 196.
Hart, John I., 239.
Harvey, H. F., 84.
Hays, F. T., 96.
Head, Joseph, 160, 162.
Heckard, W. A., 387.
Heidbrink, J. A., 249.
Heise, O. N., 391.
Hetrick, F. O., 594.
Higgins, W. J., 414.
Hinkins, Dr., 159.
Hoff, N. S., 280, 363, 369, 547.
Honey, E. A., 337.
Hoyer, F. F., 586.
Hugenschmidt, A., 161.
Hulick, W. O., 143.
Hungerford, C. L., 241, 456, 462, 466.
Hunter, F. A., 119.
Jackson, V. A . 550.
James, L. M., 25.
Jones, L. C., 270.
Johnson, C. N., 90, 324, 404.
Junkerman, G. S., 142.
Kesler, B. L., 343, 487.
Kirk, E. C., 305, 485, 561.
Kirke, H. M., 181, 185.
Lee, B. H., 584.
Litch, W. F., 548.
Logan, W. H. G., 394, 458, 462, 544.
Loeffler, E. J., 360.
Luckie, S. B., 538.
McFarland, S. S., 486.
Marshall, J. S., 467, 544, 550.

Mitchell, W. H., 376.
Mitchell, L. J., 539.
Molyneaux, G., 178.
Moore, E. C., 163.
Naumann, H. F., 269.
Newkirk, G., 549.
Noble, C. C., 290.
Nones, R. H., 106.
Noyes, E., 466.
Nyman, J. E., 384, 529.
Oakman, C. H., 290.
Olliver, R. T., 560.
Ottensen, I., 584.
Ottolengui, R., 323.
Palmer, Jas. G., 234.
Patchin, E. L., 144.
Patterson, J. D., 452, 548, 593.
Payne, D. H., 214.
Peck, A. E., 377.
Perry, S. G., 267, 268, 269, 299, 323, 473.
Peters, C. J., 107.
Pierce, C. N., 466.
Potter, W. H., 432.
Price, W. V. A., 44, 145, 370, 475.
Prinz, H., 266.
Prothero, J. H., 147.
Pruyn, C. P., 461.
Raymond, E. H., 214.
Redmond, C. E., 529.
Reese, E. E. 473.
Reeves, W. T., 232.
Rhein, M. L., 237.
Roberts, F. H., 266, 378.
Rowley, C. R., 617.
Ruggles, S. D., 47, 88, 183, 474.
Runyan, A. C., 332, 411.
Sage, F. W., 363, 404.
Semans, H. M., 140.
Shaw, J. W., 1.
Smith, B. H., 559.
Smith, H. T., 44, 86.
Smith, H. A., 63, 69, 362, 399.
Smith, J. K., 143.
Smith, R. C., 215.
Stephan, J., 120, 368.
Stephan, 583.
Stubblefield, J. A., 549.
Taggart, W. S., 216, 229.
Talbot, E. S. 455.
Taft, J., 89, 194, 341, 363, 401, 412, 448.
Truman, Wm. H., 302.
Truman, James, 457, 465.
Wakefield, R., 213.
Walton, Dr., 139.
Warboys, C. H., 334, 338, 413.
Watling, J. A., 340, 412.
Wessels, Dr., 267.
Whinnery, C. T., 144.
Whitslar, W. H., 55, 69, 88, 365, 527.
White, S. M., 332, 335.
Williams, J. Leon, 238.
Wilson, Geo. H., 68, 69, 168, 183.
Wright, C. M., 46, 66, 109, 122, 217, 362, 401, 415, 440.
Van Orden, L., 213.
Vernon, J. B., 585.
Vignes, C. V., 369, 584.
Von Beust, T. F., 199.
Young, J. L., 290, 403.
Younger, W. J., 538.

